# Partner age differences and associated sexual risk behaviours among adolescent girls and young women in a cash transfer programme for schooling in Malawi

**DOI:** 10.1186/s12889-018-5327-7

**Published:** 2018-03-27

**Authors:** Roxanne Beauclair, Jonathan Dushoff, Wim Delva

**Affiliations:** 1 International Centre for Reproductive Health, Ghent University, De Pintelaan 185, 9000 Ghent, Belgium; 2 The South African Department of Science and Technology-National Research Foundation (DST-NRF) Centre of Excellence in Epidemiological Modelling and Analysis (SACEMA), Stellenbosch University, 19 Jonkershoek Road, Stellenbosch, 7600 South Africa; 3 Department of Biology, McMaster University, 1280 Main St. West, Hamilton, ON L8S 4K1 Canada; 4 Center for Statistics, Hasselt University, Campus Diepenbeek, Agoralaan Building D, 3590 Diepenbeek, Belgium; 5 Rega Institute for Medical Research, KU Leuven, Herestraat 49, 3000 Leuven, Belgium

**Keywords:** Malawi, Sexual risk behaviour, Age-disparate relationships, Age-mixing, Southern Africa

## Abstract

**Background:**

Age disparities in sexual relationships have been proposed as a key risk factor for HIV transmission in Sub-Saharan Africa, but evidence remains inconclusive. The SIHR study, a cluster randomised trial of a cash transfer programme in Malawi, found that young women in the intervention groups were less likely to have had a sexual partner aged 25 or older, and less likely to test positive for HIV and HSV-2 at follow-up compared to control groups. We examined the hypotheses that girls in the intervention groups had smaller age differences than control groups and that large age differences were associated with relationship-level HIV transmission risk factors: inconsistent condom use, sex frequency, and relationship duration.

**Methods:**

We conducted an analysis of schoolgirls in the Schooling, Income, and Health Risk (SIHR) study aged 13-22 at baseline (*n* = 2907). We investigated the effects of study arm, trial stage and participant age on age differences in sexual relationships using a linear mixed-effects model. Cumulative-link mixed-effects models were used to estimate the effect of relationship age difference on condom use and sex frequency, and a Cox proportional hazard model was used to estimate the effect of relationship age difference on relationship duration. We controlled for the girl’s age, number of partners, study group and study round.

**Results:**

Girls receiving cash transfers, on average, had smaller age differences in relationships compared to controls, though the estimated difference was not statistically significant (− 0.43 years; 95% CI: -1.03, 0.17). The older the participant was, the smaller her age differences (− 0.67 per 4-year increase in age; 95% CI: -0.99, − 0.35). Among controls, after the cash transfers had ended the average age difference was 0.82 years larger than during the intervention (95% CI: 0.43, 1.21), suggesting a possible indirect effect of the study on behaviour in the community as a whole. Across treatment groups, larger age differences in relationships were associated with lower levels of condom use, more frequent sex, and longer relationship durations.

**Conclusions:**

Cash-transfer programmes may prevent HIV transmission in part by encouraging young women to form age-similar relationships, which are characterised by increased condom use and reduced sex frequency. The benefits of these programmes may extend to those who are not directly receiving the cash.

**Electronic supplementary material:**

The online version of this article (10.1186/s12889-018-5327-7) contains supplementary material, which is available to authorized users.

## Background

“Age-mixing” and its effect on HIV transmission dynamics is an important research area in the quest for an AIDS-free generation [1, 2]. Age-mixing patterns – how individuals in a population choose partners with regards to age – influence HIV transmission in modelling studies [3–5] and may partially explain variation in epidemic size in sub-Saharan Africa [6]. Evidence of increased individual-level risk is mixed, with studies showing a positive association of relationship age differences with HIV prevalence [7–10], though not with HIV incidence [11–13].

There are at least a few causal pathways by which younger partners may confer lower risk for HIV infection. First, age-similar young male partners are more likely to have been sexually active for a shorter period of time, and thus be less likely to be HIV positive [8, 14–16]. Second, older male partners are more likely to be in a concurrent relationship [7, 13, 17], which increases the likelihood young women will be sexually active with a man when he experiences acute HIV infection. Finally, age-asymmetric relationships have been reported to result in inconsistent condom use [18–22] and higher frequencies of sex [20, 21]. This could be due to the gender-power imbalances that may be more likely in age-disparate relationships, and which result in less ability of younger women to negotiate when and how sex occurs [21, 23].

Given these causal mechanisms, large relationship age differences are likely to increase women’s risk of HIV infection. We believe the failure to detect a consistent relationship between age differences and HIV infection by previous studies may be partially related to how data has been analysed. Until recently, empirical age-mixing studies have analysed observational data in two primary ways. The first way involves categorizing the most recent, or primary relationship into 2 or 3 categories: typically, the relationship is “non-age-disparate”, “age-disparate” (where the male partner is 5 or more years older than the female partner), and sometimes “inter-generational” (the male partner is 10 or more years older) (e.g. [12, 13]). In the second type of analysis linear correlations between relationship age differences (as a continuous variable), and HIV infection risk or prevalence are analysed (e.g. [11]). In both types of analyses relationships in which the women are older than their partners are typically discarded. However, recent studies suggest that the effect of relationship age difference on prevalent HIV infection is nonlinear, as well as age-specific [24–26].

An example demonstrating this complexity was recently published from a study on Likoma Island in Malawi [24]. They found that women who were approximately 2–12 years younger than their partners had higher probabilities of being HIV positive than women in more age-similar partnerships, but those who were more than 12 years younger than their partners had lower probabilities of being HIV positive [24]. Additionally, a phylogenetic study of individuals from a high-HIV-prevalence population in South Africa found that girls and young women aged 15-25 years old were most likely infected by men who were in their 30s, while those same men were mostly infected by women aged 25-40 years [25]. These studies indicate a need to move beyond the current paradigm and utilize analytical techniques that allow for flexible, nonlinear relationships between variables. Arbitrary categorizations and forced linear relationships may mask the true underlying risk patterns [27–30].

Recently, several structural and behavioural interventions have aimed to increase school attendance in young African girls, improve long-term economic outcomes, and thus indirectly reduce HIV transmission [9, 31–33]. A cash transfer programme in Zomba, Malawi – the Schooling, Income, and Health Risk (SIHR) study – found that the prevalence of HIV and HSV-2 was lower among 13-22 year-old girls in the intervention arm, compared to the control group, at their 18-month follow-up [34]. The intervention arm also had a lower prevalence of relationships with men 25 years or older. Some studies have suggested that young women are often motivated to engage in relationships with older men because the men may provide them with pocket money or gifts [35–38], money for school fees [39], and food [40]. The findings from the SIHR study do not necessarily mean that the lower partner ages in the intervention groups directly caused a reduction in HIV transmission, but they do suggest the hypothesis that young women receiving cash transfers may have had less motivation to choose older partners who would put them at increased risk of HIV infection, through the mechanisms identified above.

In the Malawian context, where HIV peaks in men at older ages than in women [14, 16], interventions that inhibit relationships with older men may be key to reducing incidence among adolescent girls and younger women. Using flexible nonlinear models, and publicly available data from the SIHR study, we assessed evidence for the hypothesis that smaller age differences in the intervention groups was a driver of their lower observed STI prevalences. To do this, we investigated the effect of the intervention on age differences, as well as, examined the association between age differences and relationship-level characteristics that affect HIV transmission risk: condom use, sex frequency, and relationship duration.

## Methods

### Study design and data source

We conducted a secondary analysis of the SIHR study data that comes from Zomba, Malawi. Zomba district includes one of the four largest cities in Malawi (Zomba City), in addition to a large rural population [41]. At the time of the SIHR study, it was primarily agricultural, with most people participating in subsistence farming [42], and only 6% of the adult population having a formal income as of 2008 [41]. HIV/AIDS was, and still is, one of the largest public health problems facing the district [43]. The SIHR study was a cluster randomised trial of a cash transfer programme administered to girls 13-22 years old [34]. Only aspects relevant to the present analysis will be discussed here.

Enumeration Areas (EAs) in the Zomba district were randomly sampled (*n* = 176) from urban, rural, and near-urban areas. Households with at least one never-married girl aged 13-22 years old were included in the study. Eighty-eight of the EAs were randomised to the intervention group, and the other 88 to the control group. Girls in both groups were classified as schoolgirls if they were enrolled in school at baseline, and otherwise as dropouts. Schoolgirls within intervention EAs were randomly assigned to receive: unconditional cash transfers (UCTs); conditional cash transfers (CCTs), paid only if they attended school at least 80% of the days school was in session; or no cash transfers (Spillovers). The study design included Spillovers in order to see if there would be an indirect effect of the trial on girls in intervention areas who did not receive cash. One hypothesis is that the spillover girls may also change their behaviour, because they see other girls (i.e. the ones receiving cash transfers) changing their behaviour [44]. Please see Baird et al. for the trial profile from the original study [34]. Cash was administered monthly to both households and girls in the intervention groups.

**Fig. 1:**
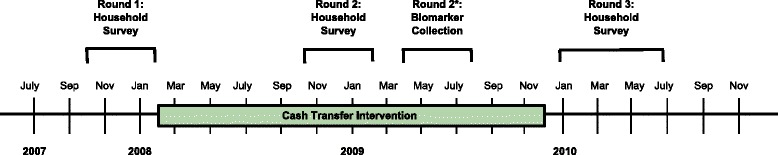
Overview of SIHR study intervention and data collection rounds. *HIV data collected during Round 2 biomarker collection were not made publicly available, and therefore, not used in our analysis

Data were publicly available for three rounds of data collection: Round 1 (baseline) took place before allocation of intervention assignment; Round 2 (R2) was during the intervention; and Round 3 (R3) was after the end of the intervention (Fig. 1; [45]). The survey was administered in two parts. The first part was administered to the heads of households and obtained information on household characteristics. The second part was administered to the participating girls and solicited responses about their health, dating patterns, and social networks.

### Participants

At baseline 2907 schoolgirls were sampled and interviewed. Our focal outcome was the age difference in a relationship, which was only recorded during R2 and R3. In each of those rounds girls could report up to 3 partners that they had in the past 12 months. Since our focus was age differences, we studied only schoolgirls who reported sexual relationships in the past 12 months at either R2 or R3. It is a shortcoming of our study that we do not have partner ages in R1, and therefore cannot control for random differences in place before the study started, nor can we detect changes in behaviour between R1 and R2. Despite this, we believe the changes that occur between R2 (during the intervention) and R3 (after the intervention) are still informative and provide insight into what policy makers and researchers might expect at the conclusion of a similar intervention. The majority of girls did not report a relationship in either round, and thus, we were left with 1108 schoolgirls who reported on a total of 1491 relationships in R2 and R3 combined. Relationships were the unit of observation in our analyses.

### Statistical analysis

Our analyses focus on four outcome variables potentially affecting HIV transmission risk: 1. *age difference* (continuous, one-year increments); 2. *condom use* (ordinal, Never/Inconsistent/Every time); 3. *sex frequency* (ordinal, Once or twice only/Less than twice per month/A couple times per month/1-3 times per week/4 or more times per week); and 4. *relationship duration* (continuous, one-week increments). We chose these variables because the probability that a girl will become infected with HIV by a sex partner is largely determined by the frequency of unprotected sex within that relationship. Inconsistent condom use, high numbers of sex acts in a given period of time, and long relationship durations, are relationship characteristics that may increase the opportunities for HIV transmission, and they may act as causal mediators in the relationship between age differences and HIV risk.

Age difference was calculated by subtracting the girl’s age from the age of her partner. This variable was used as the outcome in our first model, and then as an explanatory variable in subsequent models. Relationship duration was determined by asking girls how long ago the relationship began and how long ago the relationship ended. Relationships that were ongoing at the time of the survey were treated as having right-censored durations.

To investigate the effects of study arm, girl age, and survey round (i.e. during versus after the intervention) on relationship age difference, we fitted a linear mixed-effects regression model. Prior to constructing our model, we chose three planned contrasts for the study arm comparisons: pooled UCTs/CCTs versus controls, UCTs versus CCTs, and spillovers versus controls. We defined the pooled UCTs/CCTs to be the average of the two effects. Our primary interest was the contrast between the combined intervention groups and controls, because we wanted to see if the intervention would have an effect on choosing partner ages, regardless of conditionality. We hypothesized that intervention group girls would have smaller age differences with their partners compared to the control group during R2 when monthly cash transfers were taking place. We believed they would be less motivated to choose an older partner who might provide them with pocket money, because they would have their own source of income.

Additionally, we decided to compare spillovers versus controls to see if there was an indirect effect (cash was not received by spillovers) of being in an intervention EA. We also included a study arm-by-survey round interaction term in our model.

In two additional models, we treated condom use and sex frequency as response variables and used cumulative-link mixed effects models (CLMMs) to assess the relationship between each of these outcomes and age difference. CLMMs are commonly used when the outcome of interest is ordinal. The underlying assumption is that the effect of the covariates on the log odds of moving to a higher response level is the same at each response level. To assess the effect of age difference on relationship duration, while accounting for censorship, we used a Cox proportional hazards model.

Since sexual behaviours of young women are expected to change through time as they get older, and this effect could be conflated with effects of intervention timing, we adjusted for the participant’s age in our models. We also controlled for the number of partners in the previous 12 months, study group and study round, in the models where condom use, sex frequency, and relationship duration were outcomes, because each of these variables could have potentially confounded the relationship between age differences and the sexual behaviours. In all of the mixed-effects models we had random intercepts for the EA and girl. In the Cox model, we used robust estimators of EA-level error in calculating confidence intervals. Survey sample weights were not used in these analyses since we were not concerned with estimating population-level descriptive statistics [46].

As discussed above, flexible nonlinear models allow us to investigate a variety of risk patterns when age differences and age are continuous covariates in models. To determine the functional form of these variables in the models for condom use, sex frequency and relationship duration, we first fitted generalized additive models (GAMs) with penalized regression smoothers for the continuous variables, while adjusting for model covariates. GAMs are semiparametric models that allow continuous variables to ‘speak for themselves’ without imposing a specific form [47]. The estimated Effective Degrees of Freedom (EDF) for the continuous term was then rounded to the nearest integer and used to determine how many degrees of freedom (DF) to use for our spline terms in the final models. In the CLMM for condom use we implemented a spline for age difference with DF equal to 3 and kept age as a linear term (equivalent to a spline with 1 DF). In the CLMM for sex frequency, the spline terms for age difference and age of girl had DFs equal to 4 and 2, respectively. In the Cox model age difference had 2 DFs and age was kept as a linear term. Splines are difficult to interpret using model coefficients; we therefore used effects plots to visualize how the outcomes of interest varied as a function of the predictors. All statistical analyses were performed with R version 3.3.1 [48].

## Results

### Description of key variables

Overall, there were 1491 relationships reported: 541 in R2 (36.3%) and 950 in R3 (63.7%). Controls reported 783 relationships (52.5%), spillovers 315 relationships (21.1%), CCTs 275 relationships (18.4%), and UCTs 118 relationships (7.9%).

**Fig. 2:**
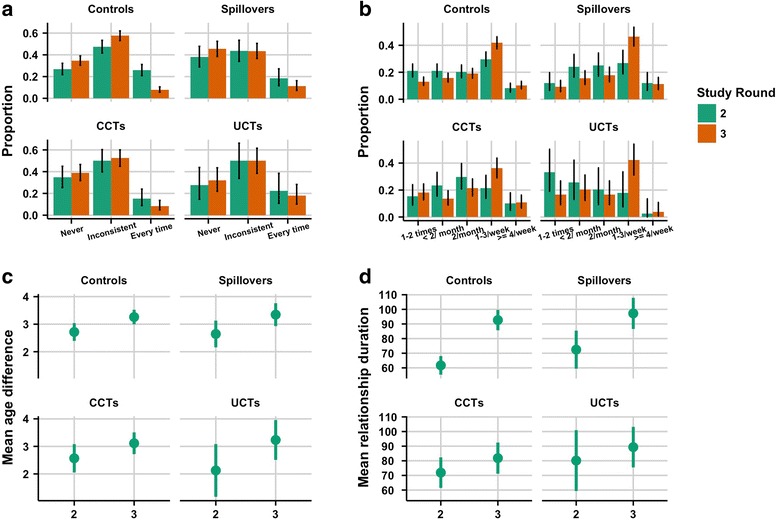
Summary statistics for relationship characteristics, by study group and round. The panels contain summaries for: **a**. condom use (*n* = 1491); **b**. sex frequency (*n* = 1490); **c**. age difference (*n* = 1364); and **d**. relationship duration (*n* = 1256)

Figure 2a shows that in all study groups and rounds there are larger proportions of the girls using condoms “Never” or “Inconsistently” than using “Every time”. The fraction of girls who reported using condoms “Every time” decreased from R2 to R3 among all study groups; this was most pronounced among the controls. In all study groups the fraction of girls who reported sex 1-3 times per week increased from R2 to R3, with the greatest jump among the UCTs: from 18.0% to 42.3% (Fig. 2b). Sex four or more times per week was relatively uncommon in all study groups. Figure 2c shows that average age differences increased from R2 to R3 in all groups. The average relationship duration increased from R2 to R3 for all study groups (Fig. 2d).

### Effects of SIHR on relationship age differences

**Fig. 3:**
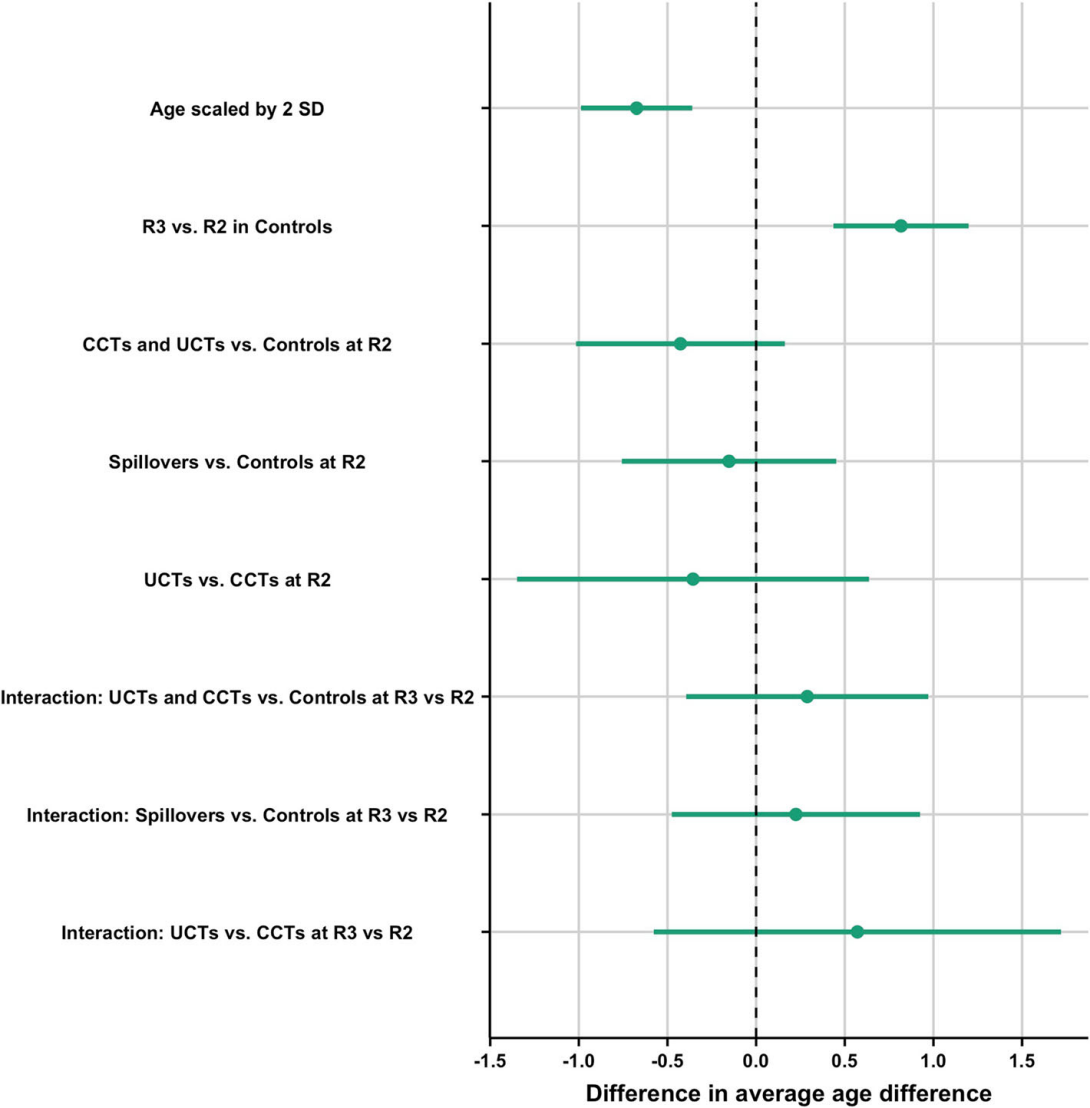
Results of linear mixed-effects model with age difference as the outcome. Beta coefficient and 95% Confidence Interval (95% CI) plot for the (fixed) effects of age, study group, and round on age difference between a girl and her partner

Results of our linear mixed-effects model (Fig. 3) show that for approximately every four years (2 SD = 3.96 years) increase in age of the girl, the average age difference decreased by 0.67 years (95% CI: -0.99, − 0.35). The average age difference was 0.82 years (95% CI: 0.43, 1.21) larger among control girls reporting in the post-intervention period (R3) compared to during the time of the intervention (R2). The observed effect of study group on average age difference was in the hypothesized direction with CCTs and UCTs having a smaller difference than control girls (− 0.43 years; 95% CI: -1.03, 0.17), though the effect was not statistically significant. The overall pattern of effects can also be seen in the Additional file 1: Figure S1.

### Condom use model

**Fig. 4:**
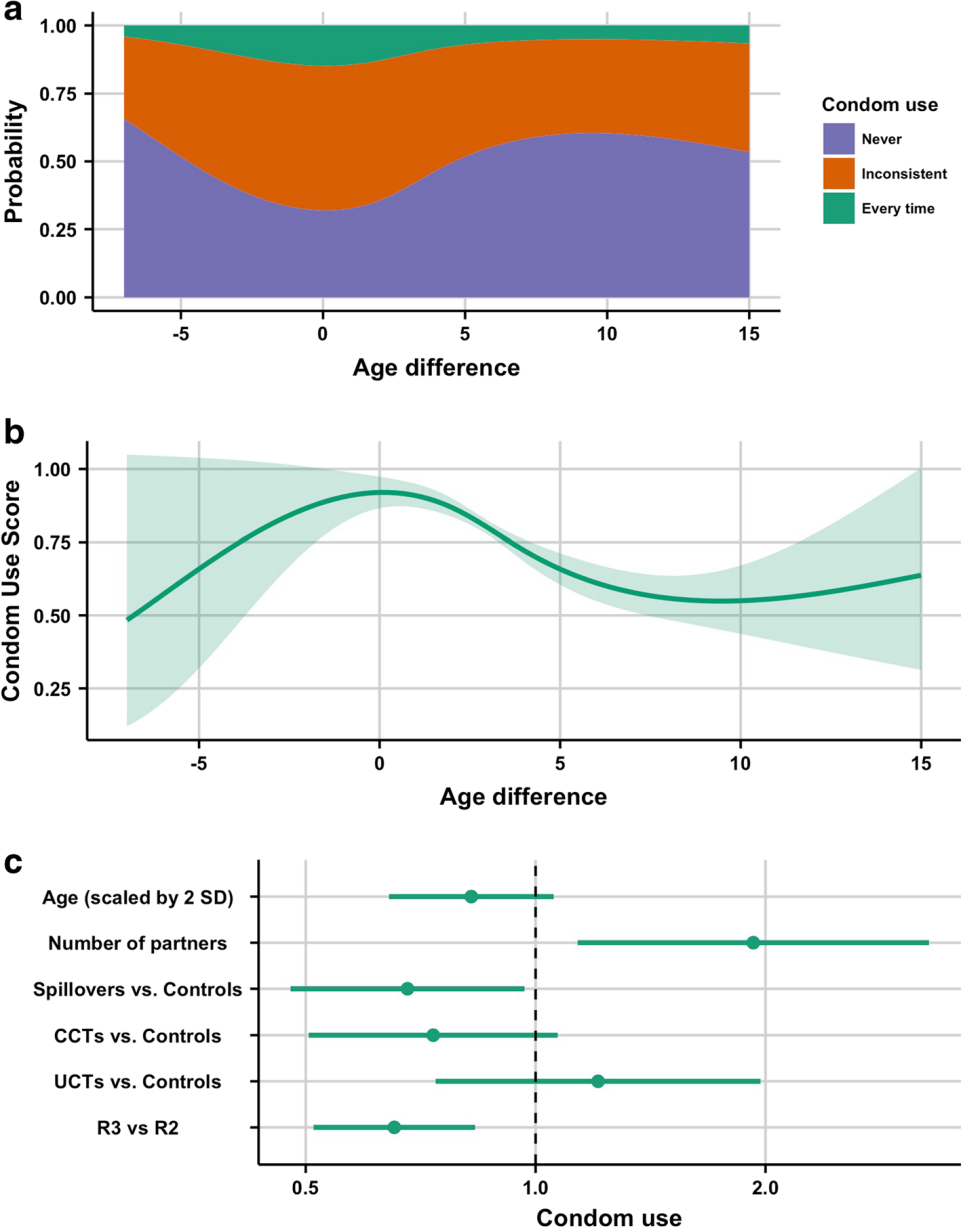
Results of cumulative-link mixed model with condom use as the outcome. In this model age difference was a spline term. **a**. cumulative probability of condom use categories for age difference. **b**. predicted effect of age difference on ordinal condom use score (scored as 0 for “never” up to 2 for “every time”), with shaded areas representing the 95% CI. **c**. proportional odds ratio (POR) and 95% CI plot for non-spline terms in the model

Figure 4a shows that the probability of using a condom “Every time” decreased as the absolute value of age difference between the girl and her partner increased. Figure 4b shows overall condom use decreased from equal-age partnerships through to an age difference of about 7 years. Patterns outside this range cannot be inferred clearly, because the CIs are large. Figure 4c shows that the odds of higher versus lower categories of condom use decreased by 18% (POR: 0.82; 95% CI: 0.64, 1.06) for approximately every four years increase in girls’ age. In R3 compared to R2, girls had 35% (POR: 0.65; 95% CI: 0.51, 0.83) lower odds of reporting more condom use. In spillovers versus controls, girls had lower odds (POR: 0.68; 95% CI: 0.48, 0.97) of higher levels of condom use. A similar, but non-significant, effect was observed in CCTs versus controls.

### Sex frequency model

**Fig. 5:**
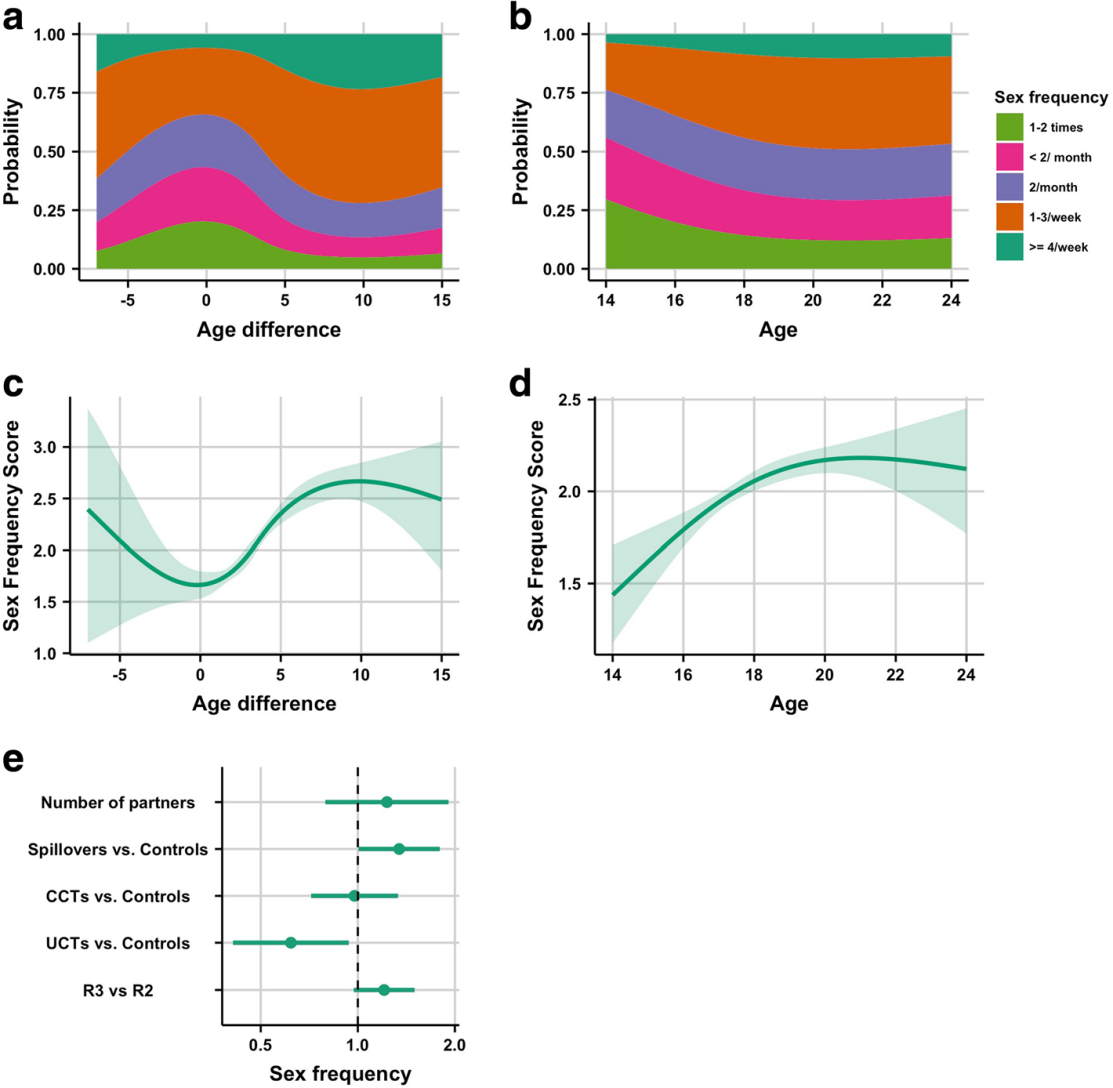
Results of cumulative-link mixed model with sex frequency as the outcome. Both age and age difference were spline terms in this model. **a**. cumulative probability of sex frequency categories for age difference. **b**. cumulative probability of sex frequency categories for age of participant. **c**. predicted effect of age difference on ordinal sex frequency score (scored as 0 for “1-2 times” up to 4 for “4 per week”) for age difference, with the shaded areas representing 95% CIs. **d**. ordinal sex frequency score for age of participant with the shaded areas representing 95% CIs. **e**. proportional odds ratio (POR) and 95% CI plot for non-spline terms in the model

Figure 5a and c suggest a nonlinear relationship between age differences and increasing sex frequency, although at the extreme values of age difference the CIs are large (5c). When girls were of similar age as their partners sex frequency tended to be low and then increased rapidly with increasing age difference until the point where partners were around 7 or 8 years older, after which it stabilized. With regards to age (Fig. 5b and d), when girls were young they tended to have a lower sex frequency. The probability that a girl had sex more frequently versus less frequently increased until around the age of 19, and thereafter remained relatively constant. Spillovers had higher odds of more frequent sex than controls (POR: 1.34; 95% CI: 1.00, 1.80), while UCTs had smaller odds of more frequent sex than controls (POR: 0.62; 95% CI: 0.41, 0.94). All girls had 1.21 times the odds (95% CI: 0.97, 1.50) of reporting more frequent sex after the intervention, compared to during the intervention.

### Relationship duration model

**Fig. 6:**
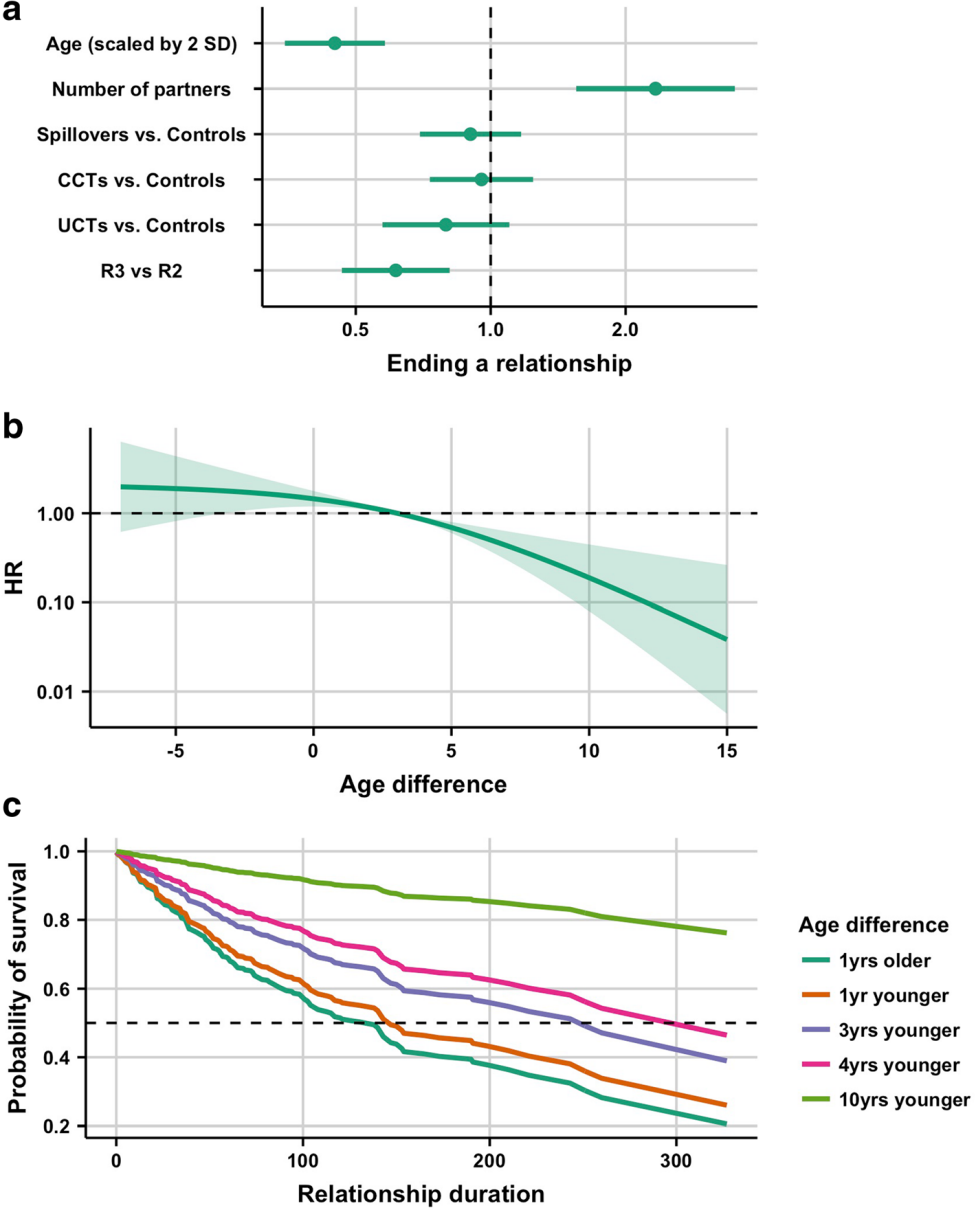
Results of Cox proportional hazards model for relationship duration. In this model age difference was represented with a spline. **a**. coefficient plot of hazard ratios for ending relationships (HR and 95% CI) for all non-spline terms in the model. **b**. predicted HRs for age differences, with the median (age difference = 3) as the reference. **c**. expected survival curves for selected age differences (2.5th, 25th, 50th, 75th and 97.5th percentiles)

Figure 6a indicates that for every 4 years increase in age, the hazard of ending relationships decreased by 55% (HR: 0.45; 95% CI: 0.35, 0.58). The hazard of ending a relationship was 39% lower (HR: 0.61; 95% CI: 0.47, 0.81) for relationships reported in R3 compared to R2. There is no convincing evidence of an effect of the study group on the hazard of ending a relationship. Figure 6b shows that the hazard of ending a relationship was greater for girls more than 3 years older than their partners compared to girls who had the median age difference (3 years younger). This hazard gradually decreased as the age differences became larger. The median relationship duration was shortest for girls one year older than their partners and longest for girls who were 10 years younger than their partners (Fig. 6c).

## Discussion

The SIHR study found that the prevalence of HIV and HSV-2 was lower among adolescent girls and young women in the intervention arms of a community randomised cash transfer trial [34]. We found little support for our hypothesis that this result might be explained by differences in partner age between arms. Although we found that reported age differences in the combined intervention groups were on average smaller than in the control group during the intervention (R2), the effect was small and not statistically significant.

This contrasts with a study of government-administered social grants in South Africa, which found that the incidence of age-disparate relationships was 71% lower among girls who were part of households receiving grants [31]. The differing findings between the study presented here and the one in South Africa might be partially explained by the differences in the way we operationalised age differences: while we used a continuous response, they dichotomized their response into those having or not having partners five or more years older than the participant. Moreover, there may have been different underlying motivations for entering age-disparate relationships in the two contexts. If schoolgirls in Zomba do not choose partners based upon financial reasons, then they would not have been incentivized to choose younger partners during the intervention, as we hypothesized.

In our study, large age differences in relationships were also associated with other behaviours potentially suggestive of heightened transmission risk, including lower levels of condom use, more frequent sex, and longer relationship durations. Our findings, along with the observation that the HIV prevalence among men in Malawi increases with increasing age up to 45 years old [16], support the hypothesis that age-disparate relationships provide more exposure to STIs, including HIV, for young women in Malawi.

While similar associations between age-disparate relationships and condom use [18–22] or sex frequency [20, 21] have been noted in other studies, the relationship between age differences and duration of relationships has been less studied. We found that the older a man was compared to the girl, the less likely they were to terminate their relationship. Longer relationships may confer more risk of HIV transmission compared to shorter ones, even if condom use and sex frequency within the relationship were held constant, because there would be more time to transmit HIV. However, such effects could be negated if girls with shorter relationships had more partners in a given amount of time. Further study of the interaction between relationship duration, partner-turnover rate, and age differences in different age groups is needed to see whether advantages of short duration in age-similar relationships are offset by choosing to have more sexual partners.

Interestingly, when the post-intervention period (R3) was compared to the intervention period there were larger age differences, less consistent condom use, more frequent sex, and longer relationship durations. If the intervention was causing the young girls who received cash to participate in safer sexual behaviours, we would expect the riskier behaviours to increase when the girls stop receiving money. However, for the study groups not receiving money, we would expect the risky behaviours to remain constant during and after the cash transfer programs, because they did not have the monetary incentives to choose safer partners. We hypothesize that this could be explained by the presence of the Hawthorne Effect [49]: because the controls and spillovers were aware of an ongoing study in their district they may have modified, or reported, their behaviour in a socially desirable way during the intervention. This effect has also been suggested to explain increased school attendance among the control communities of the South African HPTN 068 trial, measuring HIV incidence in young women in rural South Africa [50, 51].

We also observed that older girls tended to have smaller partner age differences. Others have previously found that age differences between young women and their partners remain relatively constant as they age, usually varying between 4 and 7 years depending on the setting [18, 24, 52]. While this is different from what we found, it demonstrates how age-mixing patterns are context-specific, and there may be different motivations and informal rules governing these choices from one setting to the next [53]. We also noted that older girls tended towards less condom use, and were less likely to end relationships. Moreover, they had sex more frequently (although this trend plateaued in the young adults). One potential reason for our findings might be that as girls get older, men of similar age are more likely to have ways of earning money, thus making them more attractive partners. Also, as they enter more stable relationships we would expect growing trust within the relationship, thus leading to more regular sex and less consistent condom use [54].

This study has some limitations. The first is that there may be under-reporting of relationships due to social desirability bias. We observed that only 1108 out of 2907 schoolgirls reported a sexual relationship in either R2 or R3, with only 355 girls reporting a partner in both rounds. Face-to-face interviewing methods have been shown to result in under-reporting in sexual behaviour surveys [55–57]. The effect of this bias on results, however, depends on whether unreported relationships differ systematically from those reported. Secondly, since most women did not report more than one relationship, we had limited ability to study partner turnover rate and multiple partner concurrency, both of which have been correlated with age-disparate relationships [7, 13, 17]. Furthermore, some of the relationships reported in R2 and R3 may have been with the same partner, but the survey design did not allow for unique identification of relationships. We addressed this to the best of our ability by using random-effects models to account for potentially correlated relationships data reported by participants. Finally, there were some missing data on our key variable, age difference. Fortunately, only 8.5% of the relationships (127/1491) had missing data on this variable, and therefore we do not think the magnitude of the bias would be very large.

## Conclusions

Our analysis suggests that the primary mechanisms through which age-similar relationships prevent HIV in this population may be through increased condom use, lower sex frequency and a lower background HIV prevalence among male partners. Relationship duration may also play a role. Though we did not observe a conclusive study group effect on the age differences themselves, the increase in partner ages after the programme ended compared to during the program is suggestive of potential Hawthorne effects. Thus, the HIV prevention benefits of the cash transfer intervention may have extended to those who were not receiving cash, since they also showed reduced prevalence of risky behaviours during the intervention. However, these effects, across all study groups, may be transient and only effective while cash transfers are taking place.

The quest for effective and cost-efficient interventions that prevent young women from acquiring HIV is ongoing. This analysis should encourage sustained action from policy makers in the health, education, and economic sectors to help create supportive socio-economic environments that facilitate safer relationship choices among young women while at the same time improving education levels and household income in communities where poor education, poverty and STI/HIV infection are pervasive challenges.

## Additional file

Additional file 1: **Figure S1.** Interaction plot based upon Fig. 3 from the manuscript. (PDF 267 kb)

## Abbreviations

β: β-coefficient
CCT: Conditional cash transfer
CI: Confidence interval
CLMM: Cumulative link mixed model
DF: Degrees of freedom
EA: Enumeration area
EDF: Effective degrees of freedom
GAM: Generalised additive model
HIV: Human immunodeficiency virus
HR: Hazard ratio
HSV-2: Herpes simplex virus type 2
POR: Proportional odds ratio
R1: Round 1
R2: Round 2
R3: Round 3
SIHR: Schooling, income, and health risk study
STI: Sexually transmitted infection
UCT: Unconditional cash transfer
