# Supplementary material for: Partner age differences and associated sexual risk behaviours among adolescent girls and young women in a cash transfer programme for schooling in Malawi
Source: BMC Public Health. 2018 Mar 27;18:403. doi: 10.1186/s12889-018-5327-7 (PMC5872581; doi:10.1186/s12889-018-5327-7)
Supplement: Supplementary file 1 — Figure S1. Interaction plot based upon Fig. 3 from the manuscript. (PDF 267 kb) [file 12889_2018_5327_MOESM1_ESM.pdf]

Additional file 1: Figure S1. Interaction plot based upon Figure 3 from the manuscript

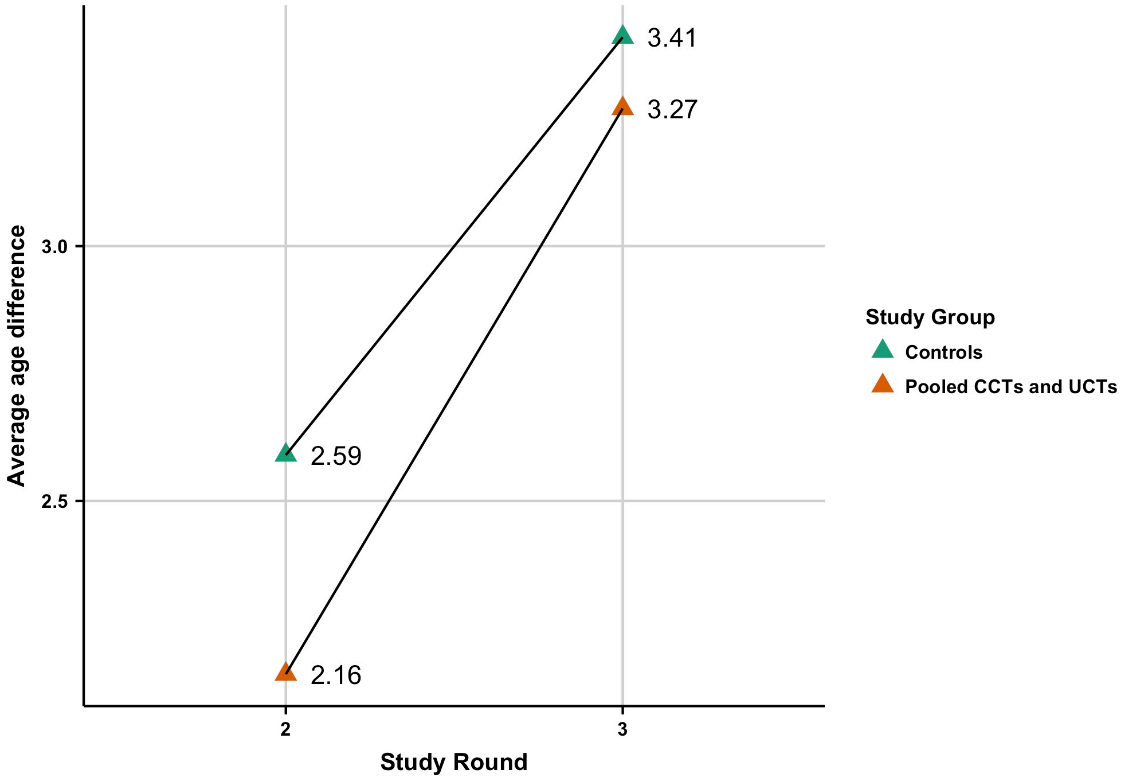

This is an example interaction plot that demonstrates how to interpret the model effects for Figure 3 in the presence of interaction terms. For the example, we use the comparison between pooled CCTs and UCTs versus the Controls. The average age difference for Controls at R2 is the intercept (Average age difference = 2.59) of the model presented in Figure 3 of the main manuscript. The average age difference for Controls at R3 is the intercept plus the estimate for the time effect (2.59 + 0.82). The average age difference for pooled CCTs and UCTs at R2 is the intercept plus the estimate for CCTs and UCTs vs Controls at R2 (2.59 - 0.43). Finally, the average age difference for the pooled CCTs and UCTs at R3 is the addition of the intercept, the estimate for CCTs and UCTs vs Controls at R2, the estimate for the time effect, and the estimate for the interaction term (2.59 - 0.43 + 0.82 + 0.29).
